# Takotsubo Syndrome in Patients with COVID-19: a Systematic Review of Published Cases

**DOI:** 10.1007/s42399-020-00557-w

**Published:** 2020-10-06

**Authors:** Sandeep Singh, Rupak Desai, Zainab Gandhi, Hee Kong Fong, Shriya Doreswamy, Virmitra Desai, Anand Chockalingam, Puja K. Mehta, Rajesh Sachdeva, Gautam Kumar

**Affiliations:** 1 Department of Clinical Epidemiology, Biostatistics and Bioinformatics, Amsterdam University Medical Center, AMC, Amsterdam, Netherlands; 2 Division of Cardiology, Atlanta VA Medical Center, Decatur, GA USA; 3 Department of Internal Medicine, Geisinger Community Medical Center, Scranton, PA USA; 4 Division of Cardiology, UC Davis Medical Center, Sacramento, CA USA; 5 Vydehi Institute of Medical Sciences and Research Centre, Bangalore, India; 6 University of North Texas Health Science Center, Fort Worth, TX USA; 7 Division of Cardiology, University of Missouri Health Care, Columbia, MO USA; 8 Division of Cardiology, Emory University School of Medicine, Atlanta, GA USA; 9 Division of Cardiology, Morehouse School of Medicine, Atlanta, GA USA; 10 Division of Cardiology, Medical College of Georgia, Augusta, GA USA

**Keywords:** COVID-19, SARS-CoV-2, Takotsubo syndrome, Takotsubo cardiomyopathy, Apical ballooning syndrome, Broken heart syndrome

## Abstract

Takotsubo syndrome (TTS) is caused by catecholamine surge, which is also observed in COVID-19 disease due to the cytokine storm. We performed a systematic literature search using PubMed/Medline, SCOPUS, Web of Science, and Google Scholar databases to identify COVID-19-associated TTS case reports and evaluated patient-level demographics, clinical attributes, and outcomes. There are 12 cases reported of TTS associated with COVID-19 infection with mean age of 70.8 ± 15.2 years (range 43–87 years) with elderly (66.6% > 60 years) female (66.6%) majority. The time interval from the first symptom to TTS was 8.3 ± 3.6 days (range 3–14 days). Out of 12 cases, 7 reported apical ballooning, 4 reported basal segment hypo/akinesia, and 1 reported median TTS. Out of 12 cases, during hospitalization, data on left ventricular ejection fraction (LVEF) was reported in only 9 of the cases. The mean LVEF was 40.6 ± 9.9% (male, 46.7 ± 5.7%, and female, 37.7 ± 10.6%). Troponin was measured in all 12 cases and was elevated in 11 (91.6%) without stenosis on coronary angiography except one. Out of 11 cases, 6 developed cardiac complications with 1 case each of cardiac tamponade, heart failure, myocarditis, hypertensive crisis, and cardiogenic shock in 2. Five patients required intubation, 1 patient required continuous positive airway pressure, and 1 patient required venovenous extracorporeal membrane oxygenation. The outcome was reported in terms of recovery in 11 (91.6%) out of 12 cases, and a successful recovery was noted in 10 (90.9%) cases. COVID-19-related TTS has a higher prevalence in older women. Despite a lower prevalence of cardiac comorbidities in COVID-19 patients, direct myocardial injury, inflammation, and stress may contribute to TTS with a high complication rate.

## Introduction

Takotsubo syndrome (TTS, takotsubo cardiomyopathy, stress cardiomyopathy, or “broken heart syndrome”) is characterized by acute left ventricular dysfunction usually in the setting of physical or emotional stress [1]. Conditions of acute stress leading to catecholamine surge have been suggested as pathophysiological mechanisms to date [2]. According to the World Health Organization, the current ongoing COVID-19 pandemic has infected over 10 million people and has led to approximately 500,000 deaths worldwide. A high burden of acute cardiac injury (19.7–27.8%), leading to significantly high mortality, has been reported in these patients [3, 4]. COVID-19 patients with cardiovascular injury have been reported to have a high burden of underlying cardiovascular comorbidities [3, 4]. Furthermore, accumulating evidence suggests a picture of severe systemic inflammation and cytokine storm in COVID-19 patients [5]. Hyperinflammatory states could lead to acute stress and injury, evident from elevated markers of myocardial injury such as C-reactive protein, pro-calcitonin, creatine kinase, myoglobin, and N-terminal pro b-type natriuretic peptide (NT-proBNP) in these patients [6]. Emerging evidence suggests a picture of cytokine storm syndrome, resembling cytokine release syndrome, in COVID-19 patients [7]. It has been observed that cytokine release syndrome is accompanied by catecholamine surge [8], which can predispose to TTS in COVID-19 patients. However, limited data on TTS in COVID-19 patients with only a handful of case reports promoted us to systematically review the published cases and pertinent outcomes.

## Methods

We searched PubMed/Medline, Web of Science, SCOPUS, and Google Scholar until June 15, 2020 for case reports and case series using these keywords: COVID-19, SARS-CoV-2, takotsubo syndrome/takotsubo cardiomyopathy, stress-induced cardiomyopathy, and broken heart syndrome. All the published case reports included in the final analysis were in English except one in Italian. Since the number of case reports is few, we translated 1 case report in Italian [9] using Google translator. Data from the article were curated and summarized in the form of country of origin, age, and gender of the patients, their presenting complaint, any coexisting comorbidities, medical interventions during hospitalization, and their outcome. Continuous variables were presented as means ± standard deviations and categorical data as absolute values and percentages. All data extraction and descriptive analysis were performed using Microsoft Excel.

## Results

Our search identified 25 articles; 9 were excluded due to duplication, 5 were excluded because they were review articles on COVID-19, and did not report any cases with TTS. Finally, 10 articles describing 12 patients for the analysis were selected [9–18] **(**Tables 1 and 2). The mean age of the reported patients was 70.8 ± 15.2 years (range 43–87 years). Of all the reported cases, 66.6% (*n* = 8) were women and mostly elderly (*n* = 8; > 60 years, 66.6%) patients. Most of the reported cases were from Italy (50%) and the USA (25%), while Belgium, Spain, and Switzerland contributed 1 case each. Only 3 reports (25%) identified the triggering/stress event in these cases. Among 12 cases, only 2 cases had positive family contact history, 1 had no contact history, and 9 did not report any contact history.

**Table 1:** Demographics, comorbidities, and presentation of COVID-19-associated takotsubo syndrome

Author, year	Age (years), Sex (M/F)	Country	Past medical history	Cardiovascular comorbidities	Travel Hx	Contact Hx	Triggering/stress event	Presenting symptoms	Time from COVID-19 presentation/to TTS in days
Dabbagh, 2020	67 F	USA	None	Non-ischemic cardiomyopathy with LVEF (40%)	-	-	COVID-19/intubation and pericardiocentesis	Left shoulder pain, cough, SOB, worsening dyspnea and orthopnea	12
Meyer, 2020	83 F	Switzerland	None	HTN		-	The emotional stress of pandemic and respiratory infection by COVID-19	Acute chest pain, dry cough, SOB	4
Minhas, 2020	58 F	USA	Diabetes mellitus, dyslipidemia	HTN	None	Father ill with similar symptoms	-	Fever, fatigue, productive cough, diarrhea, SOB	5
Moderato, 2020	59 F	Italy	Diabetes mellitus, obesity, anxiety disorder	-HTN	-	Husband died of COVID-19 recently	-	Fever, acute dyspnea, chest pain	7
Nguyen, 2020	71 F	Belgium	Hypercholesterolemia, normotensive hydrocephalus with VP shunt	HTN	-	-	-	Dyspnea, febrile, fainting	-
Roca, 2020	87 F	Italy	Breast cancer	None	None	None	Pneumonia/SARA-CoV-2 virus	Fever, chills, fatigue, dry cough, SOB	14
Sala, 2020	43 F	Italy	None	None	-	-	-	Chest pain, dyspnea	3
Solano-Lopez, 2020	50 M	Spain	Benign mediastinal tumor	None	-	-	-	Fever, dyspnea, cough, chest pain	8
Pasqualetto, 2020	84 M	Italy	Diabetes mellitus	HTN	-	-	-	Fever, dyspnea, cough, chest pain	10
Pasqualetto, 2020	85 F	Italy	None	HTN	-	-	-	Fever, dyspnea, cough, chest pain	10
Pasqualetto, 2020	81 M	Italy	Diabetes mellitus	HTN	-	-	-	Fever, dyspnea, cough, chest pain	10
Taza, 2020	82 M	USA	Diabetes mellitus, schizophrenia	HTN	-	-	-	Fever, SOB	-

*Abbreviations*: *M* male, *F* female, *LVEF* left ventricular ejection fraction, *HTN* hypertension, *HLD* hyperlipidemia, *TTS* takotsubo syndrome, *COPD* chronic obstructive pulmonary disease, *CRF* chronic renal failure, *CAD* coronary artery disease, *SOB* shortness of breath, *CKD* chronic kidney disease, *VSD* ventricular septal defect, *PDA* patent ductus arteriosus, *PHTN* pulmonary hypertension, *(-)* data not reported

**Table 2:** Diagnostics, laboratory investigations, and outcomes of takotsubo syndrome with COVID-19

Author, year	COVID-19 Test	Chest imaging	ECG findings	Cardiac imaging	Coronary angiography	Troponin ng/ml	Complications	Mechanical circulatory/respiratory support	Treatment	Outcomes at discharge/follow-up (hospital stay; days)
Dabbagh, 2020	RT-PCR	Enlarged cardiac silhouette	Low voltage in limb leads with non-specific changes	Hypokinesia of apical and periapical wall with reduced LVEF (40%)	-	Elevated BNP Mildly raised	Cardiac tamponade	Intubation	Pericardiocentesis, hydroxychloroquine colchicine glucocorticoids	Recovery (-)
Meyer, 2020	Positive IgA	Bilateral opacities	ST elevation in precordial leads with T-wave inversion	Apical ballooning with hyperkinetic basal segment	Non-significant lesion	Elevated BNP Not reported	HF	No ventilation	Conventional HF medication	Recovery (10)
Minhas, 2020	RT-PCR	Bilateral lower lobe infiltrate	ST elevation in lead I and aVL, PR interval depression diffuse ST-T wave changes	Akinetic middle to distal anterior Anteroseptal, anterolateral, and apical segments Moderately hypokinetic middle and distal inferolateral segments Hyperdynamic basal segments with reduced LVEF (20%)	Not done	Elevated BNP Not reported	ARDS Cardiogenic shock	Intubation Venovenous extracorporeal membrane Oxygenation	Dual antiplatelet, anticoagulation, dobutamine, hydroxychloroquine but discontinued later, azithromycin	Recovery (6)
Moderato, 2020	SARS-CoV-2 positive	Multiple opacities, parenchymal consolidation	Elevated lateral ST segments Symmetric negative T waves and elongated QTc	Apex akinesia with apical ballooning with reduced LVEF (40–45%)	Significant injury-free coronary artery	Elevated BNP Not reported	Respiratory failure	-	Hydroxychloroquine, azithromycin, darunavir, heparin, beta-blockers, diuretics, and IV nitrates	Recovery (10)
Nguyen, 2020	RT-PCR	Ground-glass opacity involving 10–20% of the lung	Prolonged QTc (521 ms)	Regional wall motion abnormality unrelated to coronary lesions compatible to median takotsubo	Significant lesion on the proximal LAD and the first diagonal arteries	Elevated BNP Not reported	Hypoxemia	Mechanical ventilation	Two drug-eluting stents were placed	-
Roca, 2020	RT-PCR	Multiple patchy shadows with parenchymal thickening in both lungs	Negative T waves and repolarization phase alterations	Apical ballooning and hypokinesia of the mid-ventricular segments with reduced LVEF (48%)	Coronary angiography not done due to age of the patient	Elevated BNP Not reported	Hypoxemia	Oxygen through the face mask	Ceftriaxone Azithromycin Methylprednisolone Bisoprolol Fondaparinux	Recovery
Sala, 2020	RT-PCR	Bilateral opacities and ground-glass opacities	Mild ST elevation in V1-V2 and aVR Reciprocal ST depression in V4-V6 QT prolongation	Hypokinesia LV mid and basal segment Normal apical contraction S/O reverse TTS with reduced LVEF (43%)	CT Angio revealed no aortic dissection or PE or CAD	Elevated BNP Raised	Acute virus-negative lymphocytic myocarditis	CPAP	Hydroxychloroquine Lopinavir/ritonavir	Recovery (13)
Solano-Lopez, 2020	RT-PCR	Bilateral infiltrates and perihilar ground-glass opacities	ST segment elevation in the inferior and lateral leads	Basal segment akinesia and hypercontractility of the mid-apical segments with elevated diastolic pressure	CT Angio revealed no CAD	Elevated BNP Raised	Cardiogenic shock	-	Medical support and treatment for SARS-CoV-2	Recovery (10)
Pasqualetto, 2020	RT-PCR	Ground-glass opacity with bilateral consolidation	Diffuse negative T wave with QT prolongation	Apical ballooning with basal wall hypercontractility with LVEF (53%)	CT Angio revealed no CAD	Elevated BNP Raised	Hypertensive crisis	High flow oxygen via nasal cannula	Antiviral Hydroxychloroquine Fondaparinux Aspirin Nitroglycerine Metoprolol	Recovery
Pasqualetto, 2020	RT-PCR	Ground-glass opacity with bilateral consolidation	Diffuse negative T wave with QT prolongation	Apical ballooning with basal wall hypercontractility with reduced LVEF (30%)	No CAD on autopsy	Elevated BNP Raised	Septic shock Respiratory failure	Mechanical ventilation	Antiviral Hydroxychloroquine Fondaparinux Aspirin Inotropic support	Dead
Pasqualetto, 2020	RT-PCR	Ground-glass opacity with bilateral consolidation	Diffuse negative T wave with QT prolongation	Apical ballooning with basal wall hypercontractility with reduced LVEF (42%)	CT Angio revealed no CAD	Elevated BNP Raised	-	High flow oxygen via nasal cannula	Antiviral Hydroxychloroquine Fondaparinux Aspirin Metoprolol	Recovery
Taza, 2020	RT-PCR	-	ST-elevation in II, III, aVF	Apical ballooning with LVEF (45%)	CT Angio revealed no CAD	Not elevated BNP Not reported	Respiratory failure	Intubation	Colchicine Steroid Heparin Tocilizumab	Recovered

*Abbreviations*: *LVEF* left ventricular ejection fraction, *BNP* brain natriuretic peptide, *RVR* rapid ventricular response, *LBBB* Left bundle branch block, *BiPAP* bi-level positive airway pressure, *CHF* congestive heart failure, *RBBB* right bundle branch block, *(-)* data not reported

Out of 12 cases, cardiovascular comorbidities were reported (hypertension was reported in 8 (66.66%), diabetes in 5 (41.6%), and dyslipidemia in 2 (16.6%)). The most common presenting symptoms noticed were shortness of breath/dyspnea in 12 (100%), fever in 8 (66.6%), and chest pain in 7 (58.3%) of the cases. The time interval within the first symptom to the development of TTS was 8.3 ± 3.6 days (range 3–14 days). Out of 12 cases, chest imaging in the form of a chest x-ray or CT chest was present in 11 cases. The most common chest imaging findings were bilateral opacities in 8 (72.7%) and ground-glass opacity in 6 (54.5%) cases. Overall, 9 of the reported cases had heart failure with reduced ejection fraction on echocardiography with myocardial injury noted by elevated troponin I but no significant stenosis on coronary angiography. Only 1 patient had angiographically significant proximal LAD disease requiring two drug-eluting stents. Only 9 (75%) had abnormalities on ECG, with ST segment elevation in 6 (50%), T-wave inversion in 6 (50%), prolonged QT-interval in 6 cases (50%), and low voltage complex in 1 (11.1%) case.

Out of 12 cases, during hospitalization, data on left ventricular ejection fraction (LVEF) was reported in only 9 of the cases. The mean LVEF was 40.6 ± 9.9% (male, 46.7 ± 5.7%, and female, 37.7 ± 10.6%). Brain natriuretic peptide (BNP) was reported in 6 out of 12 cases and was found to be elevated in all the 6 cases. Out of 12 cases, in 8 cases, C-reactive protein was measured and found to be elevated in all the 8 cases (168.6 ± 71.9 mg/l). Ferritin was measured in only 2 of the cases out of 12 and was raised in both the cases (1010 ± 589.7 ng/ml). In 2 of the cases, the interleukin-6 level was measured and was found to be raised (37.5 ± 41.7 pg/ml). Troponin was measured in all the reported 12 cases and was high in 11 (91.6%). Out of 12 cases, 7 reported apical ballooning, 4 reported basal segment hypo/akinesia, and 1 reported median TTS.

Out of 12 cases, 11 (91.6%) cases reported at least one complication. Out of 11 cases, 6 developed cardiac complications with 1 case each of cardiac tamponade, heart failure, myocarditis, hypertensive crisis, and cardiogenic shock in 2. Because of respiratory failure, 5 patients were intubated and 1 patient was kept on CPAP. Furthermore, 1 patient developed septic shock. Of 12 cases, 10 (90.9%) cases showed successful recovery, while 1 did not report the outcome.

## Discussion

To our knowledge, this is the first systematic review of COVID-19 patients developing TTS. Existing literature suggests that postmenopausal females are more prone to developing TTS [19] as was observed in our report. Loss of sympatholytic effect of estrogen and increased myocardial and vascular response to beta-adrenergic receptors in post-menopausal women has been suggested as one of the several reasons for increased risk of TTS [20]. However, it remains unexplored if this mechanism also poses a greater risk of TTS among elderly women with concomitant COVID-19 infection as compared with men.

In this study, only a handful of cases reported classic triggering events like the development of TTS after physical stress like intubation and/or emotional stress pointing towards the physical or emotional stress towards the development of TTS in this COVID-19 patients [10, 11, 14]. However, a large number of cases did not report any particular triggering event. In addition to the physical impact, an emotional impact in the form of social isolation leading to anxiety and stress during this pandemic could also trigger TTS [21]. We found that more than half of the patients had a current or past history of underlying cardiovascular comorbidities including non-ischemic cardiomyopathy in 1, hypertension in 8, diabetes in 5, and dyslipidemia in 1 case [9–13, 17, 18]. Patients with these comorbidities have a high burden of pro-inflammatory cytokines like interleukin-6 (IL-6) and tumor necrosis factor-α (TNF-α) [22]. Although the exact mechanism of TTS in COVID-19 is not fully explained, a cytokine storm syndrome-like picture has been seen in COVID-19 patients [7], with a high burden of pro-inflammatory cytokines like IL-6, which was reported in 2 cases [10, 18]. The cytokine storm syndrome has been noted to be accompanied by a surge in catecholamines [8]. This surge could lead to direct catecholamine toxicity and myocardial damage leading to TTS [23]. We noticed that COVID-19 patients had raised inflammatory markers in terms of raised CRP and ferritin. Furthermore, a high burden of pro-inflammatory state in these patients could lead to coagulation abnormalities and related complications. Bernardi et al., in their case report, descried left ventricular thrombus formation in a COVID-19 patient with TTS [24].

More than half of the cases presented with the classical symptoms seen in COVID-19 including chest pain and dyspnea [9, 10, 15–18], which is also frequently seen in patients with TTS [25]. Furthermore, a modest increase in cardiac troponin and ECG changes suggestive of myocardial injury was noted in all the cases, which is commonly seen in TTS [26], and could mimic acute coronary syndrome (ACS). Furthermore, SARS-CoV-2 infection could lead to myocarditis which could be misdiagnosed as TTS [15]. However, the absence of any significant coronary lesions, in most the patients who underwent coronary angiography, makes ACS an unlikely culprit.

Besides, ventriculography demonstrated the apical ballooning in most of the cases [9, 11, 14, 17, 18], apical hypo/akinesia with or without basal hyperkinesia [9, 10, 12], basal or mid and basal segment hypo/akinesia [12, 14–16], and median TTS [13] characteristic of TTS variants. Even though most of the cases recovered and discharged successfully, more than 80% of the patients developed complications such as cardiac tamponade, heart failure, cardiogenic shock, myocarditis, hypertensive crisis, or respiratory failure. A marked decrease in the systolic left ventricular function has been reported in the acute phase of TTS [1] with a prognosis depending on the nature of triggering factors. TTS secondary to emotional factors has shown a good prognosis while TTS secondary to medical conditions or procedure has shown unfavorable short and long-term prognosis [27]. All patients with QT interval prolongation except one were on hydroxychloroquine for COVID-19 treatment. Hydroxychloroquine has arrhythmogenic potential and should be used cautiously in COVID-19 patients with a high burden of myocardial injury as it could further contribute to the risk of dysrhythmias and worse outcomes in TTS.

Some limitations warrant attention while inferring these results: first, small sample size with the only case reports with TTS triggered by COVID-19 related stress was included. Second, there is a chance for publication bias, as the more challenging cases are more likely to be reported and published; and third, the lack of generalizability as the demographics and baseline information cannot be used for outcomes of a larger population without a control group.

## Conclusions

COVID-19-associated direct myocardial injury, inflammation, and stress may account for TTS despite low cardiac comorbidities. COVID-19-related TTS is predominant in older women with a high complication rate with the majority of cases recovering successfully.
